# Uridine diphosphate (UDP)-glycosyltransferases (UGTs) are associated with insecticide resistance in the major malaria vectors *Anopheles gambiae s.l.* and *Anopheles funestus*

**DOI:** 10.1038/s41598-024-70713-y

**Published:** 2024-08-27

**Authors:** Rhiannon Agnes Ellis Logan, Julia Bettina Mäurer, Charlotte Wapler, Victoria Anne Ingham

**Affiliations:** Parasitology Department, Medical Faculty, Centre for Infectious Diseases, University Hospital Heidelberg, Heidelberg University, Im Neuenheimer Feld 324, 69120 Heidelberg, Germany

**Keywords:** Anopheles, Insecticide resistance, Uridine diphosphate-glycosyltransferases, Malaria, Vector control, Entomology, Translational research

## Abstract

Malaria remains one of the highest causes of morbidity and mortality, with 249 million cases and over 608,000 deaths in 2022. Insecticides, which target the *Anopheles* mosquito vector, are the primary method to control malaria. The widespread nature of resistance to the most important insecticide class, the pyrethroids, threatens the control of this disease. To reverse the stall in malaria control there is urgent need for new vector control tools, which necessitates understanding the molecular basis of pyrethroid resistance. In this study we utilised multi-omics data to identify uridine-diphosphate (UDP)-glycosyltransferases (UGTs) potentially involved in resistance across multiple *Anopheles* species. Phylogenetic analysis identifies sequence similarities between Anopheline UGTs and those involved in agricultural pesticide resistance to pyrethroids, pyrroles and spinosyns. Expression of five UGTs was characterised in *An. gambiae* and *An. coluzzii* to determine constitutive over-expression, induction, and tissue specificity. Furthermore, a UGT inhibitor, sulfinpyrazone, restored susceptibility to pyrethroids and DDT in *An. gambiae*, *An. coluzzii*, *An. arabiensis* and *An. funestus,* the major African malaria vectors. Taken together, this study provides clear association of UGTs with pyrethroid resistance as well as highlighting the potential use of sulfinpyrazone as a novel synergist for vector control.

## Introduction

Malaria remains a leading cause of morbidity and mortality worldwide with over 249 million cases and 608,000 deaths in 2022 alone; over 95% of which occur in the African continent^1^. The most effective methods for controlling malaria are the use of insecticide-treated bed nets (ITNs), and indoor residual spraying (IRS), which kill the *Anopheles* mosquito vector and hence prevents transmission^2^. The widespread use of the relatively few chemistries in vector control has applied a strong evolutionary selection pressure on mosquitoes, leading to the emergence of insecticide resistance^3^. The most important class of insecticides in vector control are the pyrethroids, which are used on all ITNs distributed and thus, pyrethroid resistance is widespread^1,4^. The emergence of pyrethroid resistance correlates with the stalling in malaria control efforts, exemplified by 89% of sentinel reporting sites reporting resistance to at least one insecticide class^1,5^.

Insecticide resistance (IR) is a complex phenotype, encompassing behavioural changes, cuticular thickening, sequestration and most importantly, target site mutations and increased detoxification (reviewed by Ingham et al*.*^6^). Target site mutations are produced by single-nucleotide polymorphisms (SNPs) in the coding sequence of insecticide target sites. The voltage-gated sodium channel (VGSC) has the characterised knockdown resistance (*kdr*) mutations (L1014F, L1014S)^7^, and ‘new *kdr*’ (V402L-I1527T)^8^, which reduces binding efficacy of pyrethroids and organochlorines. In addition, the *Ace-1* mutation (G119S) confers resistance to both carbamate and organophosphate classes^9^. Increased detoxification of insecticides through the upregulation of protein families that metabolise these compounds or aid their clearance are reported ubiquitously across malaria endemic countries^10–12^. The detoxification system is composed of three ‘phases’: phase I includes the direct oxidation, reduction or hydrolysis of compounds; phase II is the conjugation of a moiety; and phase III is excretion. The most important and well-studied detoxifiers for insecticide resistance are cytochrome P450 monooxygenases (CYP450s) which directly detoxify multiple insecticides through hydroxylation resulting in a product that is less hydrophobic^13,14^. Although these enzymes are the best studied, other phase I enzymes, such as carboxylesterases^15^, phase II enzymes including Glutathione-S-Transferases (GSTs)^16^ and phase III transporters such as ABC-transporters^17^ have been linked to IR.

In the last decade the increased availability of transcriptomic and whole genome sequence data is progressively allowing identification of novel transcripts and genomic regions driving IR in *Anopheles* spp. These studies have repeatedly identified the overexpression of uridine diphosphate (UDP)-glycosyltransferases (UGTs) in IR populations across Africa including: *An. coluzzii* in Nigeria, Niger, Chad, and Burkina Faso^18,19^; *An. gambiae* in Burkina Faso^20^; *An. arabiensis* in Tanzania (UGT308D1)^21^; and *Anopheles funestus* in Malawi, Cameroon, Uganda and Kenya^22,23^. In addition to constitutive overexpression, UGTs have been shown to be induced following permethrin exposure in Kenyan populations^24^, following deltamethrin exposure in *An. coluzzii*^19^ and are associated with bendiocarb and DDT-resistance in Cameroon^25,26^. Furthermore, there is evidence of selective sweeps in genomic regions containing UGTs which may confer an increase in transcript expression^12,27^. Additionally, UGT overexpression has been repeatedly reported in other insecticide resistant mosquito species, such as *Aedes* spp. and *Culex* spp.^28–33^ indicating that their role may be important across multiple vector species. Interestingly, UGTs are known phase II detoxifying enzymes, and act through increasing excretion of xenobiotic compounds by conjugating a UDP-donated glucose molecule which makes the product more hydrophilic^34^.

Unlike in mosquito control, UGTs involved in agricultural pesticide resistance have been extensively validated for their role in the detoxification of a plethora of compounds, including several utilised in malaria control. Distinct families of UGTs have been characterised in resistance towards spinosad^35^, which is recommended by the World Health Organisation (WHO) for larvicidal control of *Anopheles* spp. Furthermore, neonicotinoids, namely clothianidin and imidacloprid, both used in IRS formulations, have been shown to be detoxified by numerous UGT families in multiple species^36–39^. Strikingly, many of these UGT families confer cross-resistance between imidacloprid and pyrethroids^40,41^. Importantly for vector control, UGT-mediated resistance to chlorfenapyr, a pyrrole now widely used in ITNs^35,42–44^, and pyrethroids have been demonstrated in several agricultural pest species, with UGT families linked to lambda-cyhalothrin, bifenthrin, alpha-cypermethrin and deltamethrin resistance^40,45–50^. Additionally, there is evidence of UGTs aiding resistance towards dichlorodiphenyltrichloroethane (DDT)^45^.

With the wealth of evidence of UGT-mediated resistance to insecticides across both mosquito species and agricultural pests, this study adopts multiple methods to investigate the role of UGTs in insecticide resistance in the four major African malaria vectors: the *An. gambiae* complex and *An. funestus*. Here we show that UGT308G1, 306A2 and 302A1 are over-expressed in multi-resistant *An. gambiae* and *An. coluzzii*. We demonstrate tissue specificity of these UGTs and explore their expression post-pyrethroid exposure. Finally, we use a UGT inhibitor, sulfinpyrazone (SULF), to demonstrate that UGTs confer resistance to different pyrethroids and DDT in *An. gambiae, An. coluzzii, An. arabiensis* and *An. funestus*. Taken together, we demonstrate that UGTs play a key role in insecticide resistance in four major African malaria vectors and could be used as a target for vector control.

## Results

### Phylogenetic analysis of *An. gambiae* UGTs

UGTs are a conserved protein family found ubiquitously across arthropods; however, the number of UGTs is highly variable with order-specific gene diversification and cross-species conservation^51,52^. For example, *Aedes albopictus* has 46 UGTs and *Drosophila melanogaster* has 35 UGTs, whilst just 25 UGTs are annotated in the *An. gambiae* genome and 20 in *An. sinensis.* To explore the evolutionary relationship of these, a phylogenetic tree encompassing multiple mosquito species, *D. melanogaster* and *M. domestica* was constructed (Supplementary Fig. S1). Two large clades of *M. domestica* have expanded alongside the UGT304, 303 and 35 families in *D. melanogaster* relative to the mosquito species, indicating that this expansion likely occurred after the last common ancestor (LCA) of mosquitoes, *D. melanogaster* and *M. domestica* (~ 200MYA)^53^ and the LCA of *D. melanogaster* and *M. domestica* (~ 100MYA)^54^. There is also a substantial expansion of UGTs in mosquito species compared to *M. domestica* and *D. melanogaster*, including the UGT308, 309, and 310 families in *An. gambiae*; this expansion could indicate a derived role for these UGT families in mosquitoes.

**Fig. 1:**
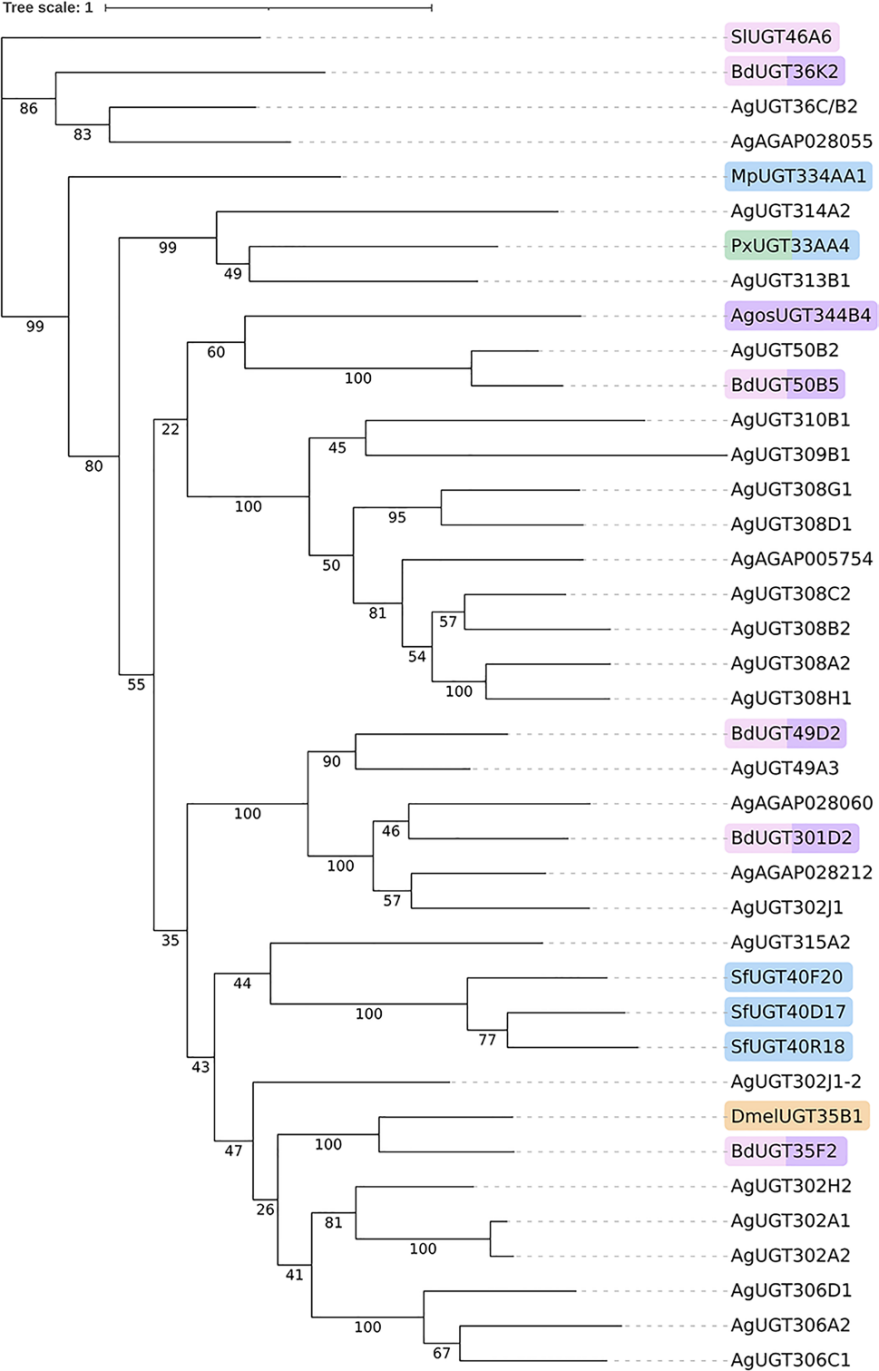
Phylogenetic tree of *Anopheles gambiae* UGTs alongside those from agricultural pests involved in resisting key malaria control insecticides. UGT peptide sequences from *Anopheles gambiae* (Ag) (25), *Bactrocera dorsalis* (Bd) (5), *Drosophila melanogaster* (Dm) (1), *Spodoptera littoralis* (Sp) (1), *Aphis gossypii* (Agos) (1), *Meteorus pulchricornis* (Mp) (1), *Plutella xylostella* (Px) (1), *and Spodoptera frugiperda* (Sf) (1) were aligned using MEGA and analysed using Maximum Likelihood method with a bootstrap of 10,000 replicates, support values are presented on each branch. UGT names start with initials of each species, family classification (numbers), subfamily classification (letters) and each UGT unique number. Anopheline gene identification numbers (AGAP) are displayed in cases with no UGT names. Tree scale indicates amino acid substitutions per site. Branches that contain resistant agricultural pest UGTs are highlighted by insecticide class: pyrroles (blue), spinosyns (green), pyrethroids (pink), neonicotinoids (purple), and organochlorines (orange).

Next, UGTs from agricultural pests that are confirmed to be involved in insecticide resistance were explored alongside *An. gambiae* (Ag) sequences (Fig. 1). UGT36C/B2 (Gene ID: AGAP007920) and AGAP028055 (no UGT name) group with *Bactrocera dorsalis* (Bd) UGT36K2 which confers cross-resistance to lambda-cyhalothrin and imidacloprid^48^. BdUGT49D2 clusters with AgUGT49A3 (AGAP007374), which is up-regulated in 29 resistance *Anopheles* transcriptomics data sets^12^, and is part of a larger cluster containing AGAP028060, AGAP028212, AgUGT302J1 (AGAP007028) and BdUGT301D2, with both *B*. *dorsalis* UGTs again causing lambda-cyhalothrin and imidacloprid resistance^48^. BdUGT50B5, also conferring resistance to lambda-cyhalothrin and imidacloprid^48^, is clustered with AgUGT50B2 (AGAP002449), and both are also related to *Aphis gossypii* (Agos) UGT344B4 which aids imidacloprid resistance in this species^38^. *Plutella xylostella* (Px) UGT33AA4 provides cross-resistance to spinosad and chlorfenapyr and groups with AgUGT313B1 (AGAP009137) and AgUGT314A2 (AGAP002783)^35^.

### Mining of -omics datasets highlights UGT differential expression

**Fig. 2:**
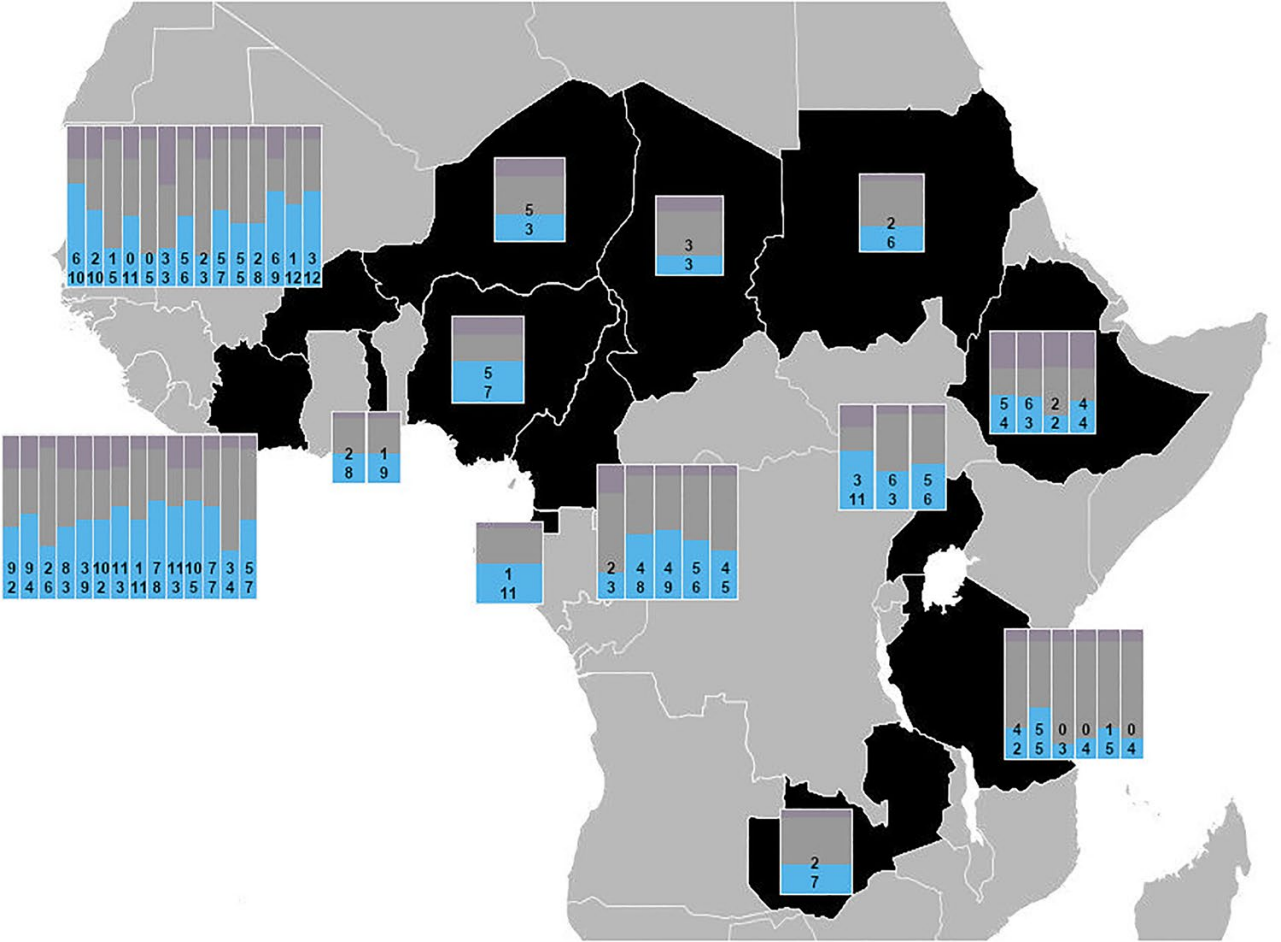
Differential expression of UGTs in published transcriptomics datasets throughout Africa. Each bar is a single dataset divided into differential expression (blue), no-significant expression (grey) and missing data (purple) for the 25 UGTs present in *An. gambiae.* Within each bar, the number of significantly upregulated UGTs are displayed above downregulated UGTs. Countries highlighted in black have available transcriptomic data: Côte D’Ivoire, Burkina Faso, Togo, Niger. Nigeria, Equatorial Guinea, Cameroon, Chad, Sudan, Ethiopia, Uganda, Tanzania, and Zambia. Data taken from Nagi and Ingham, and Ingham et al.^11,12^.

Meta-analyses of transcriptomic data have been successfully applied to identify gene families with consistent overexpression in multiple IR species^11,12^. Here, a new tool, AnoExpress, was utilised to explore the expression of UGTs in all published transcriptomic data in the *An. gambiae* species complex^12^. Mining of this data revealed significant up-regulation of at least one UGT in all 13 African countries where data is available (Fig. 2). Of particular interest is UGT302A1 (Gene ID: AGAP006222) which is one of the highest differentially expressed genes across multiple populations^12^ and is present at the foci of a selective sweep signal in West Africa within the *Anopheles gambiae* 1000 genome (Ag1000g)^27^ data. UGT302H2 (AGAP007029), UGT306A2 (AGAP007589), UGT306D1 (AGAP011564) were also chosen for further characterisation based on overexpression in the microarray (UGT302H2, UGT306A2) or RNAseq (UGT306D1) datasets. A final UGT, UGT308G1 (AGAP007990) was selected based on high overexpression in all -omics data and being reported as induced across all time points after deltamethrin exposure in an IR *An. coluzzii* population^19^.

### Transcript expression profiles of UGTs

**Fig. 3:**
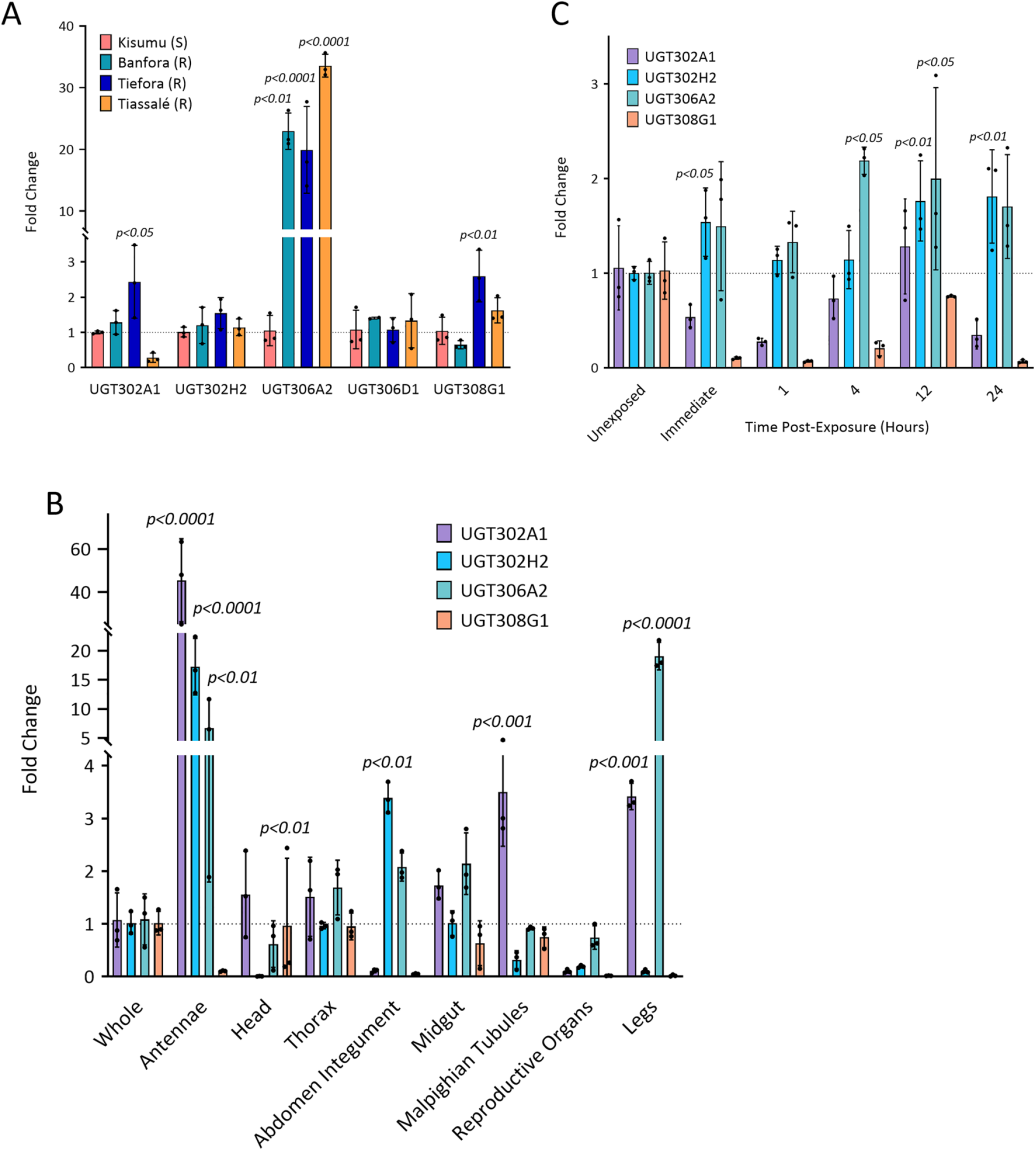
Differential UGT transcript expression using qPCR. (**A**) Three insecticide resistant populations were investigated—Banfora (dark turquoise), Tiefora (dark blue) and Tiassalé (yellow)—and compared to Kisumu (dark peach), the susceptible counterpart. The *y-axis* is the relative transcript expression fold change compared to the control, and the *x-axis* is each UGT. (**B**) Induction of UGT—UGT302A1 (purple), UGT302H2 (pale blue), UGT306A2 (pale turquoise), UGT308G1 (pale peach)—expression in *Anopheles coluzzii* at varying time points following deltamethrin exposure, each compared to the unexposed control. The *y-axis* is the relative transcript expression fold change compared to the control, and the *x-axis* is each time point that the samples were stored after exposure to the insecticide. (**C**) Tissue specificity of UGT—UGT302A1 (purple), UGT302H2 (pale blue), UGT306A2 (pale turquoise), UGT308G1 (pale peach)—expression in insecticide resistant *Anopheles gambiae* antennae, head, thorax, abdomen integument, midgut, malpighian tubules, reproductive organs, and legs, each in comparison to the whole body. The *y-axis* is the relative transcript expression fold change compared to control, and the *x-axis* is each of the mosquito tissues tested. Significance determined by one-way ANOVA and Dunnett’s multiple comparison test, thorax data was analysed by unpaired *t-test*, only statistically significant overexpression presented, dotted line represents average fold change of the control, the data are mean ± SD.

Differential expression of the candidate UGTs were firstly analysed in insecticide resistant populations of the *An. gambiae* species complex (Tiefora, Tiassalé and Banfora) and compared to an insecticide susceptible strain (Kisumu) (Fig. 3a, Supplementary Table S1). UGT306A2 was significantly overexpressed in all three resistant populations: Tiefora—19.9-fold (*p* < 0.01), Banfora—22.9-fold (*p* < 0.001), Tiassalé—33.6-fold (*p* < 0.001) (Fig. 3a). The Tiefora population also had UGT302A1 (2.4-fold, *p* < 0.05) and UGT308G1 (2.6-fold, *p* < 0.01) significantly overexpressed; however, UGT302H2 and UGT306D1 showed no overall change in expression in any population compared to the susceptible control (Fig. 3a).

Next, induction of UGTs post-pyrethroid exposure was explored. UGT expression in resistant *An. coluzzii* following 1-h exposure to 0.05% deltamethrin WHO tubes^55^ showed the induction of UGT302H2 transcripts immediately after exposure (1.5-fold, *p* < 0.05), as well as 12 h (1.8-fold, *p* < 0.01) and 24 h (1.8-fold, *p* < 0.01) post-exposure (Fig. 3b). UGT306A2 induction is delayed in comparison with an increase in expression observed at 4 h (2.2-fold, *p* < 0.05), 12 h (twofold, *p* < 0.05) and 24 h (1.7-fold, *p* < 0.01) post-exposure. Interestingly, this population showed no induction of UGT308G1. Due to the influence of the mosquito’s circadian rhythm, the expression patterns of the UGTs were investigated to identify their 12-h cycle. In parallel with the exposed mosquitoes, unexposed mRNA was isolated immediately after the deltamethrin exposure and 12 h-post exposure (Supplementary Fig. S2). Comparisons of these cohorts displayed a significant difference in expression for UGT302A1, UGT302H2 and UGT308G1 inferring that any increase in expression at 12 h post-exposure is potentially obscured by the natural decrease in expression due to circadian rhythms.

Finally, tissue specific expression of the UGTs of interest was explored. The highest expression of UGTs was observed in the legs and the antennae (Fig. 3c) which are tissues that most commonly interact with insecticide-treated surfaces. In the legs, UGT306A2 (19.1-fold, *p* < 0.0001) and 302A1 (3.4-fold, *p* < 0.001) expression levels were significantly escalated, and in the antennae UGTs 302A1 (45.5-fold, *p* < 0.0001), 302H2 (17.3-fold, *p* < 0.0001) and 306A1 (6.7-fold, *p* < 0.01) showed the most dramatic increases displaying a potential role of UGTs in olfaction. An important tissue for insecticide resistance is likely the Malpighian tubules^56^, and here, UGT302A1 transcript levels are significantly elevated (3.5-fold, *p* < 0.001). Furthermore, UGT302H2 (3.4-fold, *p* < 0.01) is elevated in the abdomen integument where metabolic homeostasis is maintained by the fat body^57^. The head of the mosquito, consisting of the proboscis, palps, and brain, displayed a significant decrease in UGT302H2 (0.0065-fold, *p* < 0.0001).

### RNAi of UGTS of interest did not restore susceptibility to pyrethroids

**Fig. 4:**
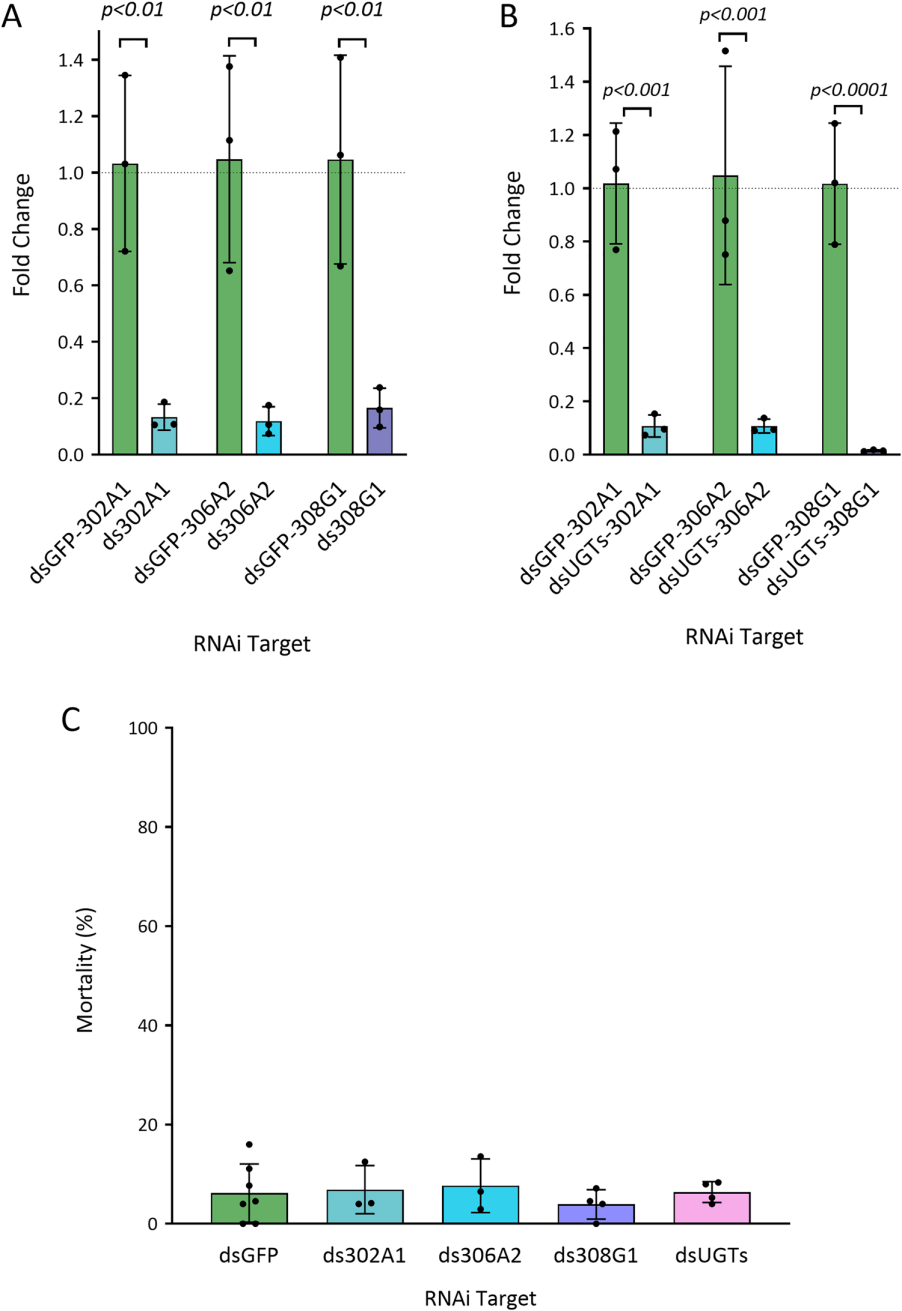
Silencing of candidate UGTs using RNAi in insecticide resistant *Anopheles coluzzii*. (**A**) dsRNA targeting individual candidate UGTs—302A1 (turquoise), 306A2 (blue), 308G1 (purple)-  efficiently reduced transcript levels when tested using RT-qPCR compared to a dsGFP (green) non-target control. (**B**) Combined dsRNA targeting the all three candidate UGTs (dsUGTs) efficiently reduced transcript levels of each when tested using RT-qPCR compared to a dsGFP non-target control. In both graphs the *y-axes* are the relative transcript expression fold change compared to dsGFP control, and the *x-axes* are the targets of the RNAi. (**C**) Silencing individual and combined candidate UGTs (dsUGTs) (pink) does not increase mortality when exposed to deltamethrin compared to a dsGFP non-target control. The *y-axis* is the 24 h mortality post-exposure, and the *x-axis* are the target of the RNAi. Statistical significance determined by unpaired *t-*test for RT-qPCR data (**A,B**) or one-way ANOVA and Dunnett’s multiple comparison test (**C**), non-significant statistics not presented, dotted line in (**A,B**) represents fold change of the control, in all cases the data are mean ± SD.

Validation of the functional role of the three most overexpressed UGTs—302A1, 306A2 and 308G1—was investigated using RNAi to determine whether targeted knockdown of these transcripts can restore susceptibility to deltamethrin in resistant *An. coluzzii* (Fig. 4)*.* The efficiency of the dsRNA was determined using qPCR; transcript levels were successfully reduced by 87% (302A1, *p* < 0.001), 88.5% (306A2, *p* < 0.001), and 80.7% (308G1, *p* < 0.001) (Fig. 4a). Despite the silencing of UGT transcripts there was no impact on the sensitivity of this population to deltamethrin (Fig. 4c). Mortality induced by deltamethrin exposure was 7.7% in the dsGFP non-target control, 6.9% in the ds302A1 group, 7.7% in the ds306A2 group, and 6% in the ds308G1 group (Fig. 4c). As the UGT family is large it is possible that targeting individual UGTs is having little effect due to functional redundancy; therefore, dsRNA for all three candidates (ds306A2 + 302A1 + 308G1) was pooled and injected. Despite the combined dsRNA efficiently silencing each of the targeted UGTs (Fig. 4b)—302A1 (reduced 89.8%, *p* < 0.001), 306A2 (reduced 89.4%, *p* < 0.001), and 308G1 (reduced 98.5%, *p* < 0.0001)- there was no increase in the sensitivity of these resistant mosquitoes to deltamethrin at only 6.4% mortality (Fig. 4c; Supplementary Table S2).

### Sensitivity of resistant Anophelines to insecticides due to UGT inhibition

**Fig. 5:**
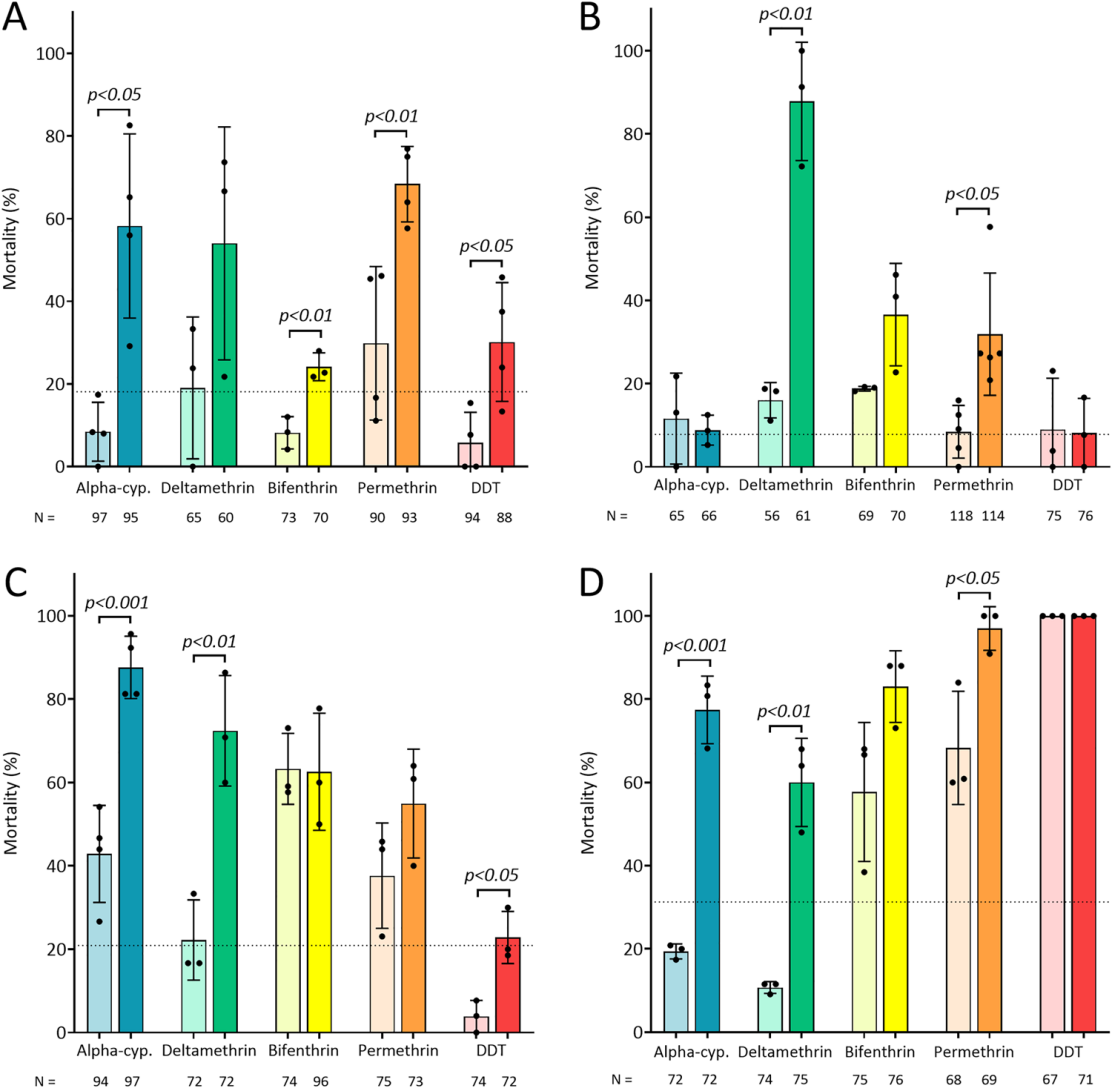
Inhibition of UGTs with sulfinpyrazone increases the sensitivity of resistant Anopheline species to an array of insecticides. (**A**) *Anopheles gambiae,* (**B**) *Anopheles coluzzii*, (**C**) *Anopheles arabiensis*, and (**D**) *Anopheles funestus*. Darker shaded bars represent mortality induced by the insecticide and sulfinpyrazone combination and lighter shaded bars represent mortality induced by the insecticide alone. Insecticides tested were alpha-cypermethrin “Alpha-cyp” (turquoise), deltamethrin (green), bifenthrin (yellow), permethrin (orange), and dichloro-diphenyl-trichloroethane “DDT” (red). The *y-axes* are mortality (%) caused by each condition, and the *x-axes* are the tested conditions. The dotted line represents mortality induced by sulfinpyrazone alone. Statistical significance determined by unpaired *t-*tests for each insecticide, non-significant statistics not presented, mosquito numbers (N =) displayed under each bar, the data are mean ± SD.

As targeting individual UGTs had no effect, the topical application of the UGT inhibitor sulfinpyrazone (SULF)^49,58^ was used to inhibit the activity of all UGTs simultaneously in four IR African species prior to insecticide exposure: *An. gambiae*, *An. coluzzii*, *An. arabiensis* and *An. funestus*. These populations all have high intensity resistance against the tested insecticides (pyrethroids: alpha-cypermethrin, deltamethrin, bifenthrin and permethrin and the organochloride DDT with 3.9–40% mortality), with the exception of *An. funestus* which is completely susceptible to DDT (Fig. 5; Supplementary Table S3). As a preliminary method of testing the impact of the inhibitor, increasing doses of SULF was topically applied^59–61^ with the resistant populations with and without WHO-tube 0.05% deltamethrin exposure to determine a concentration whereby there was minimal intrinsic mortality but clear increase in pyrethroid-induced mortality; thus, a concentration of 1% SULF was used with other insecticides (Supplementary Fig. S3, Table S3). At the 1% concentration used, there is varying levels of intrinsic mortality in the different species when SULF is applied alone (Fig. 5; Fig. S4): *An. gambiae*—18.1%, *An. coluzzii*—7.8%, *An. arabiensis*—20.9%, and *An. funestus*—31.3%.

Next, the resistant mosquito populations were treated with a topical application of 1% SULF^59^ followed by WHO-tube assays for the previously mentioned insecticides. Inhibiting UGTs in *An. gambiae* produces significant increases in mortality to alpha-cypermethrin (49.8% increase, *p* < 0.05), bifenthrin (16% increase, *p* < 0.01), permethrin (38.6% increase, *p* < 0.01), and DDT (28.1% increase, *p* < 0.05) (Fig. 5a). The increases observed for bifenthrin and DDT, however, are likely due to the additive mortality of sulfinpyrazone instead of the inhibition of UGTs, as demonstrated by the synergy ratio of close to or equal to one: bifenthrin—1.01; DDT—0.9 (Supplementary Table S5). This ratio computes the additive or synergistic impact of SULF by dividing the combined insecticide and SULF survival with a calculated survival rate, a ratio of less than one indicates a synergistic affect, equal to one is additive and more than one is antagonistic (Supplementary Table S5)^62^. *An. coluzzii* demonstrates almost complete restoration of deltamethrin susceptibility (71.8% increase, *p* < 0.01) and partial restoration of mortality towards permethrin (29.9% increase, *p* < 0.05), although the latter increase is likely due to the additive lethality of sulfinpyrazone (synergy ratio—0.81). There is a trend of increasing mortality with bifenthrin; however, this is not statistically significant and thus likely due to the intrinsic activity of sulfinpyrazone (synergy ratio—0.85) (Fig. 5b, Supplementary Table S5). The *An. arabiensis* population tested here demonstrates increasing mortality for alpha-cypermethrin (42.2% increase, *p* < 0.001), deltamethrin (50.2% increase, *p* < 0.01), and DDT (18.9% increase, *p* < 0.05) (Fig. 5c). As with *An. gambiae* it seems that the increase in mortality when exposed DDT is the additive effect of the insecticide with sulfinpyrazone (synergy ratio—1.02) (Supplementary Table S5). There is a trend of increasing permethrin susceptibility (21.2% increase; synergy ratio—0.91); however, this is not statistically significant (Fig. 5c, Supplementary Table S5). Mortality to alpha-cypermethrin (synergy ratio—0.41) and deltamethrin (synergy ratio—0.65) is again restored when inhibiting UGTs, with increases of 58% (*p* < 0.001) and 49% (*p* < 0.01) respectively in *An. funestus* (Fig. 5d). A smaller, but significant increase in mortality is also demonstrated with permethrin mortality (28.7%, *p* < 0.05; synergy ratio—0.14) after the addition of sulfinpyrazone. There is some restoration of bifenthrin susceptibility (25.6% increase; synergy ratio—0.58); however, due to the variability between replicates this is not statistically significant.

To ensure that SULF was not inhibiting the known pyrethroid metabolisers of the CYP450s, competitive binding assays were carried out with CYP6P3, CYP9K1, CYP6M2 and CYP6P4^63^. The lack of affinity of CYP450s for sulfinpyrazone ensures that the observed mortality was not induced by the inhibition of these major pyrethroid metabolisers (Supplementary Fig. S5; Table S4); however, we cannot rule out other off target effects.

## Discussion

Resistance to insecticides used in malaria control is complex, with continued reports of novel mechanisms which overcome insecticide-induced mortality^8,11,12,64,65^. Understanding such mechanisms is necessary to protect the current control tools and to innovate new chemicals and methods for implementing mosquito control. Here, we show that multi-resistant *Anopheles* species display elevated expression of multiple UGTs over time and from widespread geographical areas. We then characterise the relationship of UGTs linked to insecticide resistance in *Anopheles* with those from agricultural pests with proven roles in resistance. We further establish that key UGTs identified in transcriptomics data have increased expression of these transcripts in highly resistant lab-reared mosquitoes. Expression analysis indicates that these transcripts are enriched in tissues important for insecticide contact such as the legs and head and are induced after pyrethroid exposure. Finally, and crucially, we show that inhibition of these enzymes restores pyrethroid and DDT susceptibility in all major African vector species: *An. gambiae, An. coluzzii, An. arabiensis* and *An. funestus*.

Mining of transcriptomic datasets collected over disparate time periods demonstrates consistent overexpression of UGTs in IR populations representing all the major African malaria vectors^12,19^. UGTs found overexpressed across multiple subfamilies in *An. gambiae* cluster with UGTs from agricultural pests linked to resistance to compounds used in malaria vector control^35,47,48^. Interestingly, the UGT308 (UGT308G1 studied here), 309 and 310 family, which show expansion in mosquito species relative to *D. melanogaster* and *M. domestica,* cluster with *A. gossypii* AgosUGT344B4 which has been previously linked with neonicotinoid resistance^38^ and *B. dorsalis* BdUGT50B5 linked to neonicotinoid and pyrethroid cross-resistance^48^. Furthermore, UGT302H2, UGT306D1 and UGT306A2, three other UGTs identified in this study, cluster with *D. melanogaster* DmelUGT35B1 and BdUGT35F2, both of which have been identified in resistance to DDT, pyrethroids, and neonicotinoids, respectively^48,66^. As silencing of these individual genes did not restore susceptibility to pyrethroid insecticides whilst inhibition with SULF does, it would be of further interest to explore other UGTs clustering with resistance-related UGTs of other pest species. For example, AgosUGT344B4 and BdUGT50B2 provide neonicotinoid and pyrethroid resistance^38,48^ and cluster closely with *An. gambiae* UGT50B5. Moreover, there is an abundance of data to support the role of UGTs in pyrethroid resistance in *Chrysodeixis includens*, *Spodoptera exigua*, *Tetranychus urticae*, *A. gossypii*, *Sp. litura*, and *Sp. littoralis*^41,45–47,49,50^ which were not explored here due to lack of availability of the sequences.

Here, UGT306A2 was shown to be overexpressed in three resistant populations compared to the control and further induced upon pyrethroid exposure; this UGT has previously been shown to be up-regulated across all populations in Côte D’Ivoire and Burkina Faso^67^ and across temporally and geographically disparate *An. funestus* and *An. gambiae s.l.*^18,26,68,69^. The UGT302 family is associated with pyrethroid resistance in *B. dorsalis* as well as being enriched in the antennae and maxillary palp in this species, suggesting a role in odour transduction and detoxification^48^. The UGT302 family has also been identified as over expressed in pyrethroid-resistant *An. sinensis*^70^, and in a recently published study on *An. funestus*^22^. The UGT308 family was also differentially expressed in pyrethroid resistant *An. funestus* compared to insecticide susceptible mosquitoes^23^. UGT306A2 shows overexpression in the Tiefora population as expected^71^, as well as 308G1 and 302A1, but UGT302H2, observed as one of the mostly highly upregulated genes in the study by Williams et al.^71^, is not significantly overexpressed at a basal level in this study. The latter, however, is up-regulated post-deltamethrin exposure alongside UGT306A2 and conversely 308G1 is down-regulated. Surprisingly, UGT308G1 was previously shown to have sustained overexpression following deltamethrin exposure in *An. coluzzii*^19^, indicating population-specific differences in response to insecticide challenge.

The UGTs explored here show mixed tissue localisations; however, UGT302A1, UGT306A2 and UGT306A2 are all enriched in the antennae and legs. The localisation to these tissues is also seen in *Sp. littoralis* and *B. dorsalis* displaying resistance to various insecticides including deltamethrin, lambda-cyhalothrin, and imidacloprid^48,50^. These studies outlined a dual role of UGTs in odorant metabolism and insecticide resistance hinting that they may play a similar role in *Anopheles*. Intriguingly in situ hybridisation has highlighted UGT expression at the site of olfactory neurons in *B. dorsalis* antennal sensilla^50^, as the nervous system is the target of multiple malaria control insecticides^72^. As these tissues are likely the most important for insecticide uptake and have recently been shown to be the site of potential sequestration^64^, it may be that UGT detoxification is important in these tissues for insecticide metabolism.

In addition to leg and antennal expression, UGT302H2 and UGT302A1 are enriched in the abdomen integument and the Malpighian Tubule, respectively. Expression of UGTs in the Malpighian tubules and the abdomen, where the fat body is located, further points to the role of UGTs in insecticide resistance as these tissues help mediate the impact of xenobiotics throughout the insect body^56,57,73^. CYP450s and GSTs, known Anopheline insecticide detoxifiers, as well as UGTs, are overexpressed in Malpighian tubules of resistant *D. melanogaster*, and silencing CYP6G1, which is specific to this tissue, improves insecticide sensitivity^74–77^. A study in *Sp. exigua* found UGTs and CYP450s co-localise in the fat body of these resistant insects, and when treating fat body cells in vitro with a range of insecticides, including pyrethroids, these same enzyme families were then induced as a response^46^. These co-localisations provide some evidence of a sequential detoxification pathway of insecticides with UGTs (Phase II) further detoxifying the metabolites of CYP450s (Phase I)^78,79^. Interestingly, UGT308G1 and CYP6M2 follow the same induction patterns observed over 24 h post-deltamethrin exposure in *An. coluzzii*^19^ which could also indicate a link between these enzyme groups.

To assess the role of UGTs in resistance on a phenotypic level two methods were adopted: (i) RNAi was used to silence the UGTs, and (ii) sulfinpyrazone was used to inhibit them^39,43,49,58,80–82^. Characterising UGTs in vitro was not possible by silencing individual UGTs despite the dsRNA efficiently reducing the UGT transcripts to lower levels observed in resistant agricultural pests (*Myzus persicae, Diaphorina citri, Plutella xylostella, A. gossypii*)^39,58,80,82,83^. The lack of phenotype observed here could be due to three primary reasons. Firstly, there could be functional redundancy within the family, as observed with CYP450s upon RNAi. Secondly, the metabolites that are processed by the UGT enzymes may not be directly toxic; however, this is refuted by the SULF inhibition and finally, the UGTs targeted in this paper may not be directly involved in pyrethroid metabolism.

Despite a lack of phenotype when silencing the UGTs identified in this paper, the chemical inhibition of the enzyme superfamily restored susceptibility to pyrethroids and DDT in the *An. gambiae s.l.* and *An. funestus* strains tested, painting a potential role for UGTs in resistance to these insecticides. The consistent phenotype seen in both *An. gambie s.l.* and *An. funestus,* separated by 85 MY of evolution^12^, indicates that these enzymes are involved in insecticide metabolism. Although UGTs are unlikely to directly metabolise pyrethroids, the products of CYP450 metabolism (phase I) are reported to cause mortality in *An. funestus*^78^ and thus inhibiting this pathway with sulfinpyrazone could lead to a build-up of lethal insecticide metabolites. In addition to increased mortality after insecticide exposure, SULF resulted in varying levels of intrinsic mortality when applied alone to the different Anopheline species, and this could be due to the disruption of critical pathways such as tissue homeostasis as seen in mammals, fungi, and plants^84,85^. Furthermore, UGTs are involved in the metabolism of many endogenous compounds in insects that aid mechanisms such as steroid regulation, UV shielding, and cuticle formation^51^, and again, the interruption of these pathways could be having fatal consequences. The ability of sulfinpyrazone to produce these lethal affects, as well as providing synergistic results when alongside currently used insecticides, is integral evidence of how this compound could be used as a new vector control tool.

Although SULF restored susceptibility to pyrethroids and DDT, it did so differentially across species and across pyrethroid chemistries. The varying profiles of restored insecticide susceptibility with this inhibitor demonstrates population-specific and insecticide-specific mechanisms of resistance, as well as highlighting how mosquitoes have evolved multiple resistance mechanisms^6^. In line with results observed here, a pattern of differential response to varying pyrethroids across populations is also observed in field populations with the CYP450 inhibitor, piperonyl butoxide (PBO). Indeed, populations of *An. gambiae s.l.* from different countries, and even different ecological zones within a country, respond contrastingly to deltamethrin and permethrin, with PBO exposure restoring varying levels of partial susceptibility^86–90^. Analogous to SULF, PBO specifically binds and inhibits CYP450s and thus restores mortality in mosquitoes through blocking phase I detoxification^91^ and is currently incorporated on pyrethroid-PBO bed nets being used in Africa^1,4^. Taken together, this suggests that CYP450-mediated resistance, as with UGT-associated resistance, is population- and insecticide-dependant. Despite these differing profiles, PBO bed nets are efficiently reducing malaria burden through increased mortality in mosquito vectors, indicating that SULF could be an additional tool for vector control. Given the reduced efficacy of PBO in areas without CYP450 resistance^86,88,92^, it could be interesting to investigate the additive effects of PBO and sulfinpyrazone together, especially given the intrinsic mortality caused by sulfinpyrazone.

Inhibition of UGTs restores susceptibility to both pyrethroids and DDT, thus demonstrating that UGT-mediated resistance to these compounds is an overlooked mechanism in current studies. Future investigations to directly link enzymatic activity with pyrethroids or pyrethroid metabolites in *Anopheles* mosquitoes would be key in understanding the exact molecular basis of this resistance mechanism. The overexpression of UGTs in olfactory and detoxification tissues observed in this study hints at a potential role in olfaction and a synergy with cytochrome P450s, though further studies are needed to confirm this. Taken together, this study shows a concrete link between UGT enzymes and pyrethroid resistance, highlighting a new avenue for malaria control.

## Methods

### Mosquito husbandry and strains

Mosquitoes were reared at Heidelberg University in standard insectary conditions; 27 °C, 70–80% relative humidity, 12 h light cycle with 1 h dawn:dusk. Larvae are fed on ground fish food (Tetramin, Germany) and the adults on 10% sucrose solution. The mosquito colonies used in the experiments were the insecticide susceptible Kisumu from Kenya (*An. gambiae* s.s), and insecticide resistant populations: Tiassalé from Côte D’Ivoire (*An. gambiae* s.l), Tiefora and Banfora from Burkina Faso (*An. coluzzii*), Gaoua from Burkina Faso (*An. arabiensis*), and FuMOZ from Mozambique (*An. funestus*)^71,93^. Resistant colonies are selected to maintain resistance every fourth generation on 0.05% deltamethrin and 0.75% permethrin. All mosquitoes used for testing were presumed mated.

### Phylogenetic analysis of UGTs

To assess the evolutionary relationship of UGTs amongst Diptera, a phylogenetic tree was constructed by the Maximum Likelihood method with the Jones-Taylor-Thornton (JTT) model in RAxML-NG v. 1.1 with a bootstrap of 1000 replicates^94^. UGT peptide sequences were available at VectorBase.org and aligned in Clustal.org^95^. Sequences were from *An. gambiae* (AGAP) (26), *An. arabiensis* (AAR) (23), *An. sinensis* (ASIS) (20), *An. funestus* (AFUN) (24), *Aedes aegypti* (AAEL) (34), *Ae. albopictus* (AALFPA) (46), *Culex quinquefasciatus* (CQUJ) (34), *Drosophila melangogaster* (FBG) (35), and *Musca domestica* (MOD) (36).

For the phylogenetic tree of insecticide resistant UGTs, available putative UGT mRNA and peptide sequences from agricultural pests (*Bactrocera dorsalis* (5)^48^*, Drosophila melanogaster*^66^ (1)*, Spodoptera littoralis*^50^ (1)*, Aphis gossypii*^38^ (1)*, Meteorus pulchricornis*^42^ (1)*, Plutella xylostella*^35^ (1), and *Spodoptera frugiperda*^43^ (3) involved in resistance to compounds used in malaria vector control were identified from NCBI and Washington State University (https://t.ly/sjrkB). Putative *Anopheles gambiae* UGT peptide sequences (26) were collated from VectorBase (https://t.ly/75UHN). mRNA sequences were translated into peptide sequences, then all sequences were aligned in the Clustal Omega tool within MEGA11^95,96^. The phylogenetic tree was constructed by the Maximum Likelihood method with the Jones-Taylor-Thornton (JTT) model in RAxML-NG v. 1.1 with a bootstrap of 10,000 replicates^94^.

### Identification of candidate UGTs

Published transcriptomics data was analysed to investigate UGT expression throughout Africa and identify candidates for characterisation and differentially expressed transcripts were extracted from AnoExpress (https://t.ly/bXKfF) for the *Anopheles gambiae* species complex; this included both RNAseq and microarray data^11,12^. R (ggplot2) was then used to create a map displaying the fractions of the 25 UGTs quantifiable across all datasets and those showing differential expression. Five UGTs were regularly overexpressed throughout this data or shown to be induced across multiple time points post-insecticide exposure^97^ and were selected for characterisation—UGT302A1 (Gene ID: AGAP006222), UGT302H2 (AGAP007029), UGT306A2 (AGAP007589), UGT306D1 (AGAP011564), UGT308G1 (AGAP007990).

### RNA extraction and cDNA synthesis

For whole body RNA extractions, 3–5-day old female mosquitoes were collected in triplicate containing seven adults each. RNA extraction from specific tissues was also completed in triplicate, 10–100 individual tissues were dissected per sample from 3 to 5-day old females. Dissections of the following tissues were completed with tweezers and pins in iced PBS or extraction buffer: legs, ovaries, abdomen integument including fat body, malpighian tubules, midguts, antennae, thorax, head, and reproductive organs. All samples were homogenised in 100 μl Extraction Buffer from the PicoPure™ RNA Isolation Kit, the manufacturer’s protocol was then followed (ThermoFisher Scientific, Germany). Subsequently, cDNA was synthesised per RNA sample following the SuperScript™ III First-Strand Synthesis System protocol using Oligo(dT)_20_ primers to select for messenger RNA (mRNA) (ThermoFisher Scientific, Germany). cDNA was purified using the QIAquick PCR purification kit following the manufacturer protocol (QIAGEN, Germany). Quality and quantity of RNA and cDNA was measured using a NanoDrop One spectrophotometer (Thermo Scientific, Germany).

### Quantitative analysis of UGTs using qRT-PCR

Primers were designed using Primer Blast (NCBI) spanning exon-exon junctions for an 80–150 bp product length and 40–60% GC content (Supplementary Table S6). Quantitative real-time PCR (qRT-PCR) was performed using Brilliant III Ultra-Fast SYBR® Green qPCR Master Mix (Agilent, Germany) on a CFX96™ Real-Time System (Bio-Rad, Germany) with CFX Maestro 1.1 software (Bio-Rad, Germany). Each biological replicate was diluted to 2 ng/μl in triplicate. Relative expression was normalised against the housekeeping genes: elongation factor Tu (EF) (AGAP005128) and 40S ribosomal protein S7 (S7) (AGAP010592). Each 20 μl reaction contained 1 μl of 2 ng/μl cDNA, 10 μl 2× SYBR master mix and 0.3 μM of each primer. Each sample was completed in triplicate. The qPCR conditions were 3 min at 95 °C, with 40 cycles of 10 s at 95 °C and 10 s at 60 °C.

### Silencing of UGTs with RNA interference

Primers were designed for targets using Primer Blast (NCBI) for a 300–600 bp product length and 20–50% GC, T7 promoter sequences were added to 5ʹ end of forward and reverse primers (Supplementary Table S6). PCR products were amplified under the following conditions: 98 °C for 30 s, then 35 cycles of 98 °C for 7 s, 55 °C for 10 s and 72 °C for 60 s, and a final extension for 5 min at 72 °C. Amplicons were either PCR/gel purified using the QIAquick Gel Extraction Kit and QIAquick PCR & Gel Cleanup Kit following the manufacturer’s manual (QIAGEN, Germany). Double-stranded RNA (dsRNA) was synthesised using MEGAscript™ T7 Transcription Kit (Thermo Fisher Scientific, Germany) following the user manual with a 16 h incubation at 37 °C. Samples were purified using the MEGAclear™ Transcription Clean-Up Kit (Thermo Fisher Scientific, Germany) with a twice-heated elution step at 65 °C for 10 min in 100 μl final elution volume. The dsRNA quality and quantity were measured on a NanoDrop One spectrophotometer (Thermo Scientific, Germany) and concentrated to 3 μg/μl in a vacuum centrifuge. 3–5-day old female mosquitoes were anaesthetised on CO_2_ and 69 nl of dsRNA was injected into the thorax between cuticle plates for RNA interference (RNAi). In parallel, dsGFP was injected as a non-target control at the same concentration and volume. 72 h post-injection, the dsRNA was tested for efficiency using qPCR with transcript expression compared to the dsGFP control. Subsequent injections were followed by exposure to insecticides after 72 h. Insecticide tube assays were performed using standard WHO protocol^98^ with deltamethrin (0.05%) for 1 h, 24 h mortality was scored.

### Topical inhibition assays with sulfinpyrazone

Stocks of sulfinpyrazone (European Pharmacopoeia Reference Standard, Marck, Germany) were produced in acetone. 3–5-day old mosquitoes were anaesthetised on ice and 0.5 μl of 0% (acetone-only control) and 1% sulfinpyrazone was applied topically to each mosquito thorax in groups of 25^39,43,49,58–61,80–82,99^. After 1 h, up to 25 mosquitoes from each group were exposed to insecticide tubes following the standard WHO tube assay^98^ for 1 h: 0.2% bifenthrin (PESTANAL®, Merck, Germany), 0.75% permethrin (PESTANAL®, Merck, Germany), 0.05% alpha-cypermethrin (PESTANAL®, Merck, Germany), 4% 4,4′-DDT (PESTANAL®, Merck, Germany). Mosquitoes applied with 1% sulfinpyrazone were also exposed to WHO control tubes as a negative control. Dose–response curves of sulfinpyrazone (0%, 0.001%, 0.01%, 0.1%, 0.5% and 1%) were produced following application of increasing sulfinpyrazone dilutions and exposure to 0.05% deltamethrin WHO tubes for 1 h. Topical inhibition assays were performed in 3–5 replicates and 24 h mortality was recorded.

### Cytochrome P450 inhibition assays

The affinity of key cytochrome P450s—CYP6P3, 6P4, 6M2 and 9K1—to sulfinpyrazone was determined by inhibiting the metabolism of the fluorescent diethoxyfluorescein (DEF) substrate. Sulfinpyrazone (1000, 200, 40, 8, 1.6, 0.32, 0.064, 0.0128 μM) and DEF (5 μM) was prepared in DMSO, with a final solvent concentration of 3%. Reactions were 200 μl containing 0.1 μM CYP450 and an NADPH regenerating system of 50 mM K_3_PO_4_ at pH 7.4 containing 1 mM glucose-6-phosphate (G6P), 1 U/ml G6P dehydrogenase, 0.1 mM NADP+, 0.25 mM MgCl_2_. Negative controls were carried out in the absence of the NADPH regenerating system. Each reaction occurred in triplicate in a black, flat-based, opaque 96-well plate on a fluorescence plate-reader (Ex ¼ 485 nm, Em ¼ 520 nm), assays were monitored for 20 min after the addition of the NADPH regenerating system. Relative fluorescence units per nmol CYP450 per second (RFU/nmol/s) was calculated by linear regression of the difference in RFU between 10 and 13 min after assays were started.

### Statistical analysis

Results are presented as mean with standard deviation. All statistical analysis and graphs were produced by GraphPad Prism version 9 for Windows (GraphPad Software, La Jolla California USA, https://t.ly/SPQ3X). All data passed Shapiro–Wilk’s test for normality, qPCR, RNAi, and topical data were analysed using a one-way ANOVA and Dunnett’s multiple comparison test. dsRNA efficiency data was analysed using unpaired *t-tests*.

### Supplementary Information

Supplementary Information.

Supplementary Figures.

## Data Availability

Datasets analysed for this study are included in this article, Supplementary Information Table and Supplementary Figures. Transcriptomics data is stored in the Github repository for the AnoExpress python package—https://github.com/sanjaynagi/AnoExpress. Sequences were all available from public repositories at Washington State University (https://t.ly/sjrkB) and VectorBase (https://t.ly/75UHN).
