# Supplementary material for: Uridine diphosphate (UDP)-glycosyltransferases (UGTs) are associated with insecticide resistance in the major malaria vectors Anopheles gambiae s.l. and Anopheles funestus
Source: Sci Rep. 2024 Aug 27;14:19821. doi: 10.1038/s41598-024-70713-y (PMC11350197; doi:10.1038/s41598-024-70713-y)
Supplement: Supplementary file 2 — Supplementary Figures. [file 41598_2024_70713_MOESM2_ESM.docx]

Uridine diphosphate (UDP)- glycosyltransferases (UGTs) confer insecticide resistance in the major malaria vectors *Anopheles gambiae* *s.l* and *Anopheles funestus*

**Supplementary Figures**


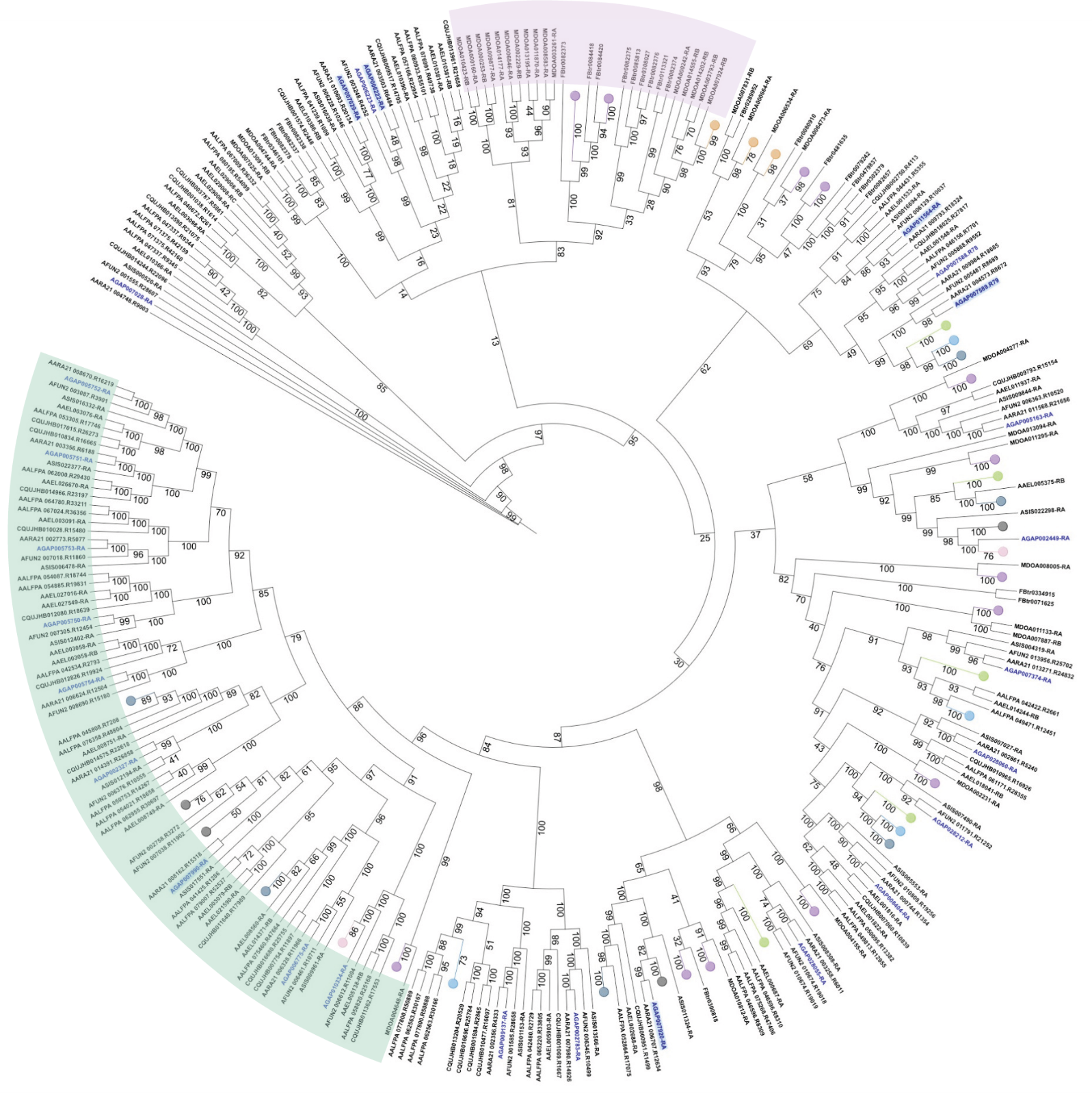


**Figure S1: Phylogeny of UGTs across Diptera.** *An. gambiae* (AGAP), blue text, those explored in more detail in this paper are highlighted in with light blue box, *An. arabiensis* (AAR), *An. sinensis* (ASIS), *An. funestus* (AFUN), *Ae. aegypti* (AAEL), *Ae. albopictus* (AALFPA), *Cx. quinquefasciatus* (CQUJ), *D. melanogaster* (FB) and *M. domestica* (MDOA) UGTs. Green shading represents an expansion of UGTs in mosquitoes, pink shading represents an expansion of UGTs in *D. melanogaster* and *M. domestica*. Splice variant ‘clades’ are represented by a circle with the following colour codes: AAR: pink; AFUN: grey; AAEL: light blue; AALFPA: dark blue; CQUJ: green; FBG: purple and MOD: orange. Bootstraps values from 1000 bootstraps shown on the branches.


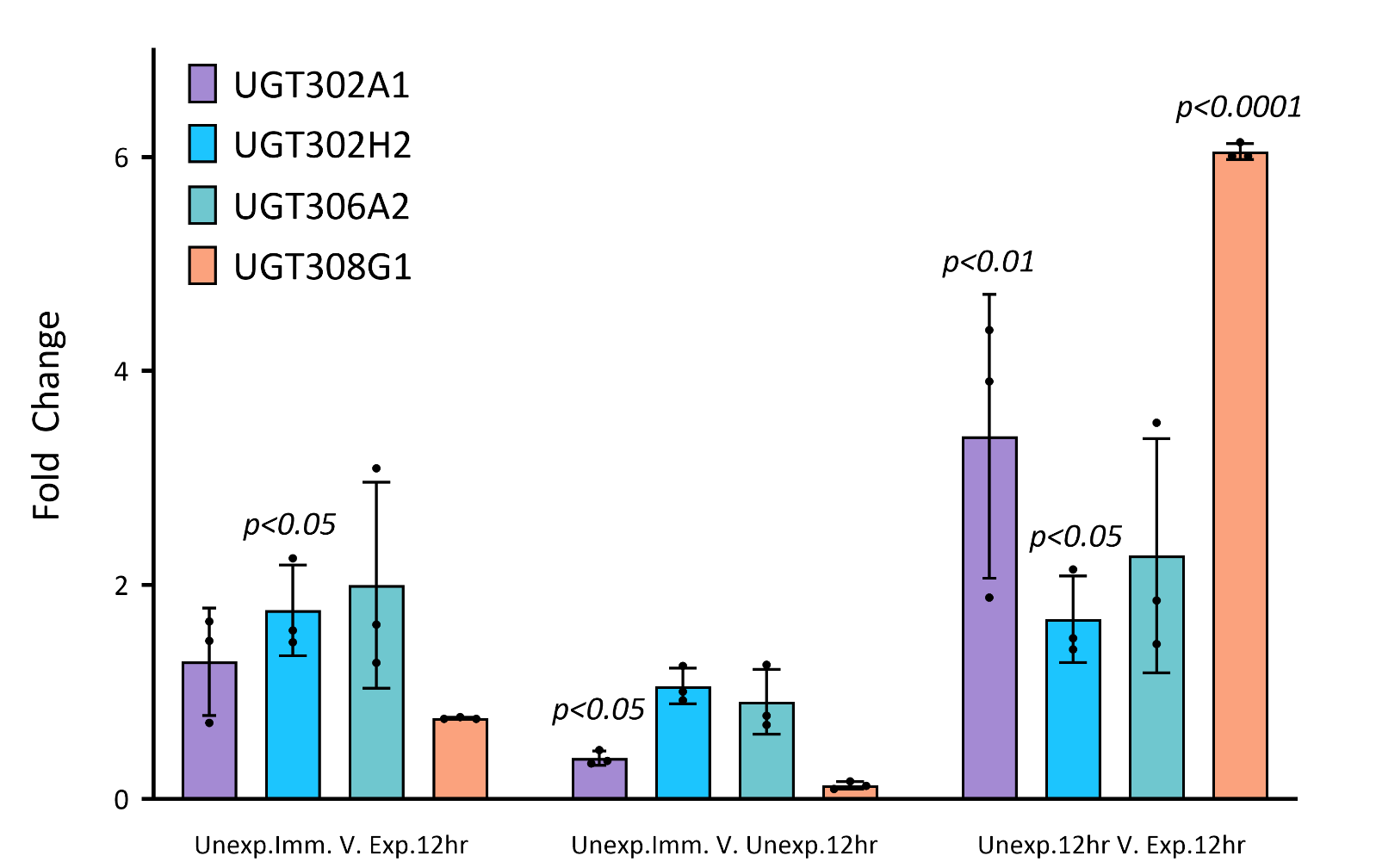


**Figure S2. Differential UGT transcript expression analysis with RT-qPCR due to circadian rhythm of resistant *Anopheles coluzzii*.** Induction of UGTs – UGT302A1 (purple), UGT302H2 (blue), UGT306A2 (turquoise), UGT308G1 (peach) – mRNA isolated from mosquitoes 12 hours post-exposure ‘Exp.12hr’ to 0.05% deltamethrin. ‘Unexp.12hr.’ is mRNA isolated from unexposed mosquitoes in parallel as the 12 hours post-exposure samples. ‘Unexp.Imm’ is mRNA isolated from unexposed mosquitoes in parallel to mosquitoes removed immediately following exposure to 0.05% deltamethrin. The *y-axis* is the relative transcript expression fold change compared to control, and the *x-axis* is each comparison. Significance determined by one-way ANOVA and Dunnett’s multiple comparison test, only statistically significant overexpression presented, the data are mean ± SD, dotted line represents 1.


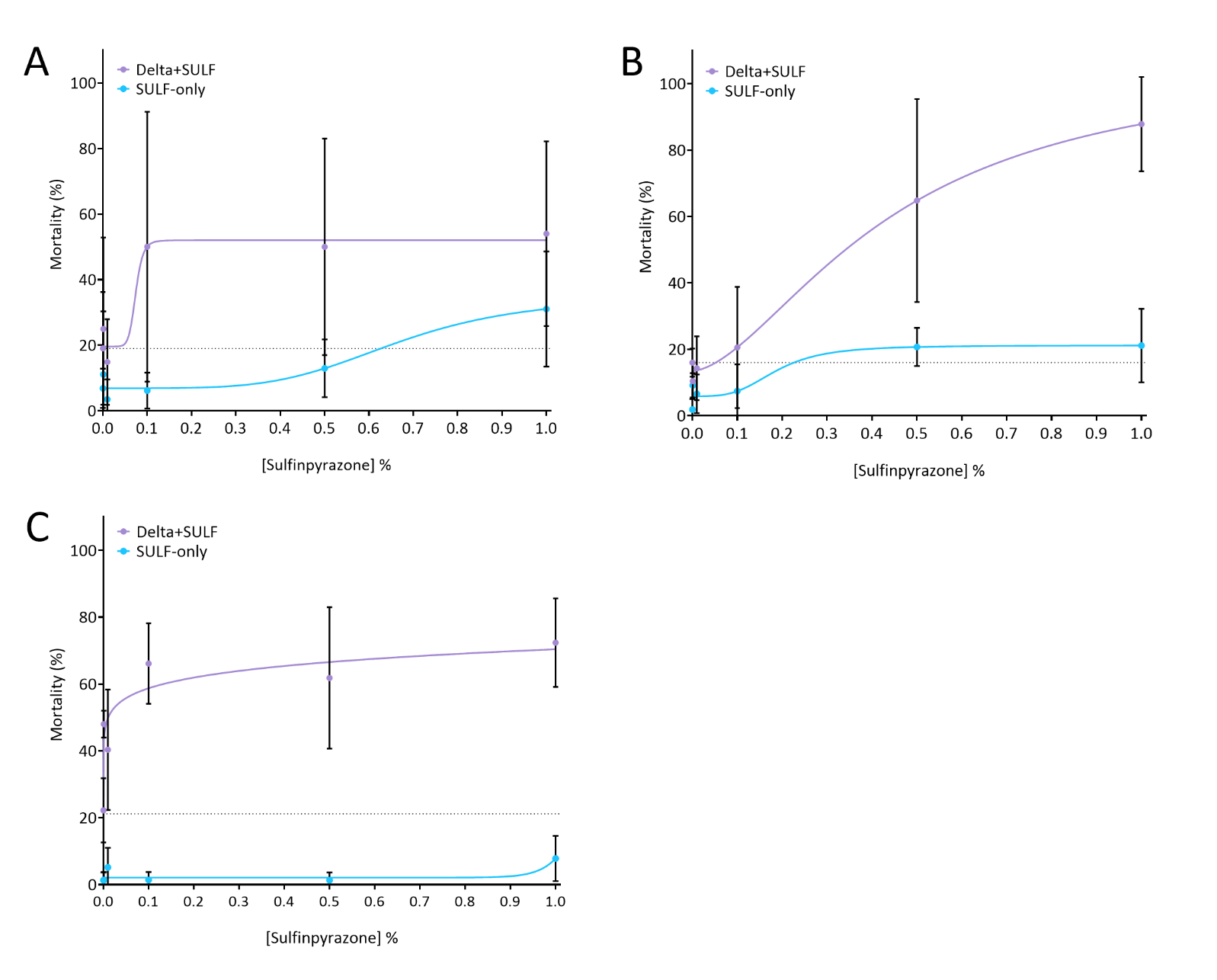


**Figure S3. Increasing sensitivity to 0.05% deltamethrin through the inhibition of UGTs by sulfinpyrazone.** (A) *Anopheles gambiae,* (B) *Anopheles coluzzii*, (C) *Anopheles arabiensis*. The *y-axis* is the mortality (%) caused by each condition, and the *x-axis* is concentration of sulfinpyrazone (%). Mortality of sulfinpyrazone alone is blue and the combination of deltamethrin plus sulfinpyrazone is purple. Dotted line represents mortality induced by deltamethrin alone, the data are mean ± SD.


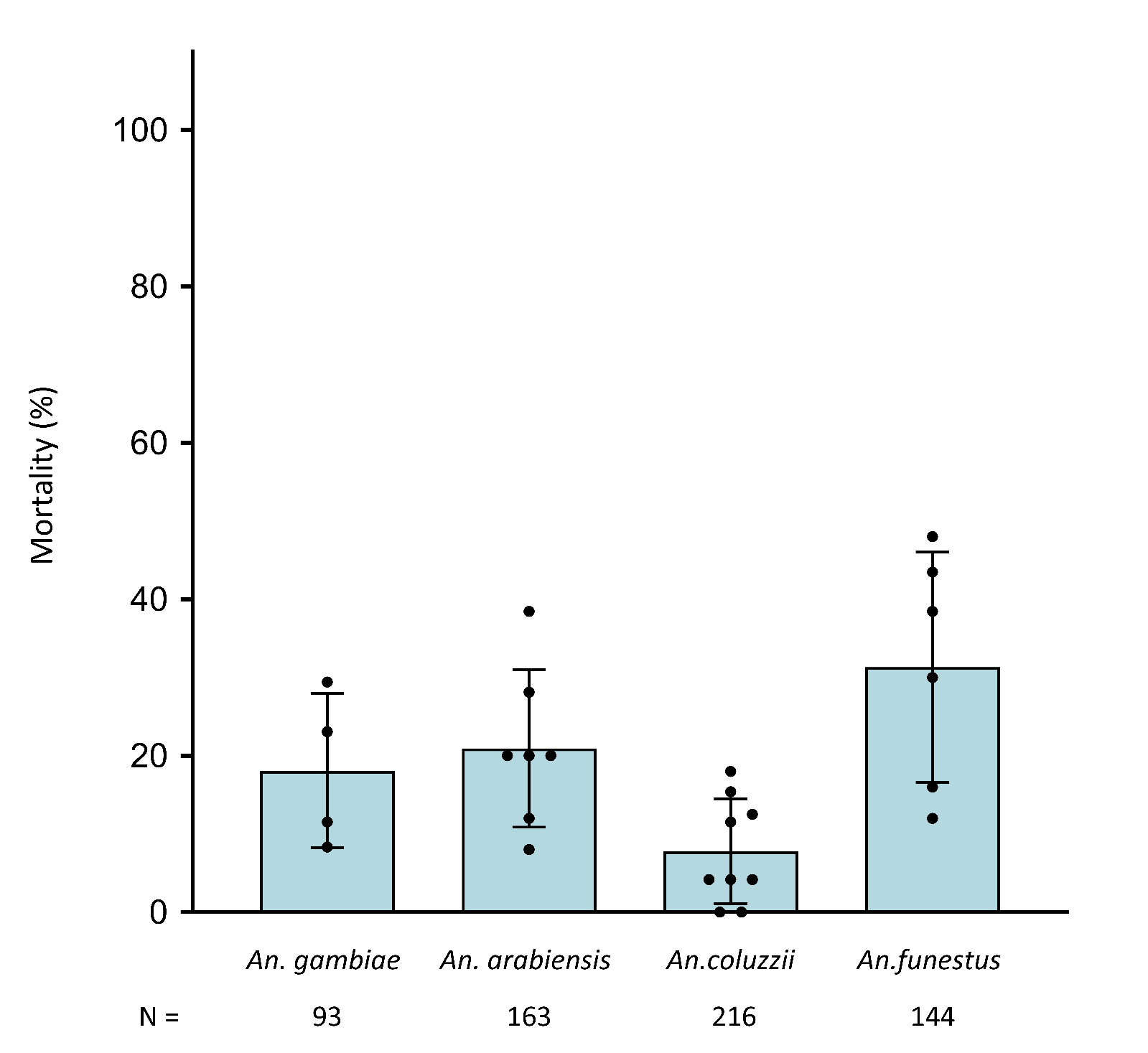


**Figure S4. Mortality caused by the topical application of 1% sulfinpyrazone in acetone.** The *y-axis* is the mortality (%), and the *x-axis* is each species tested, the data are mean ± SD.


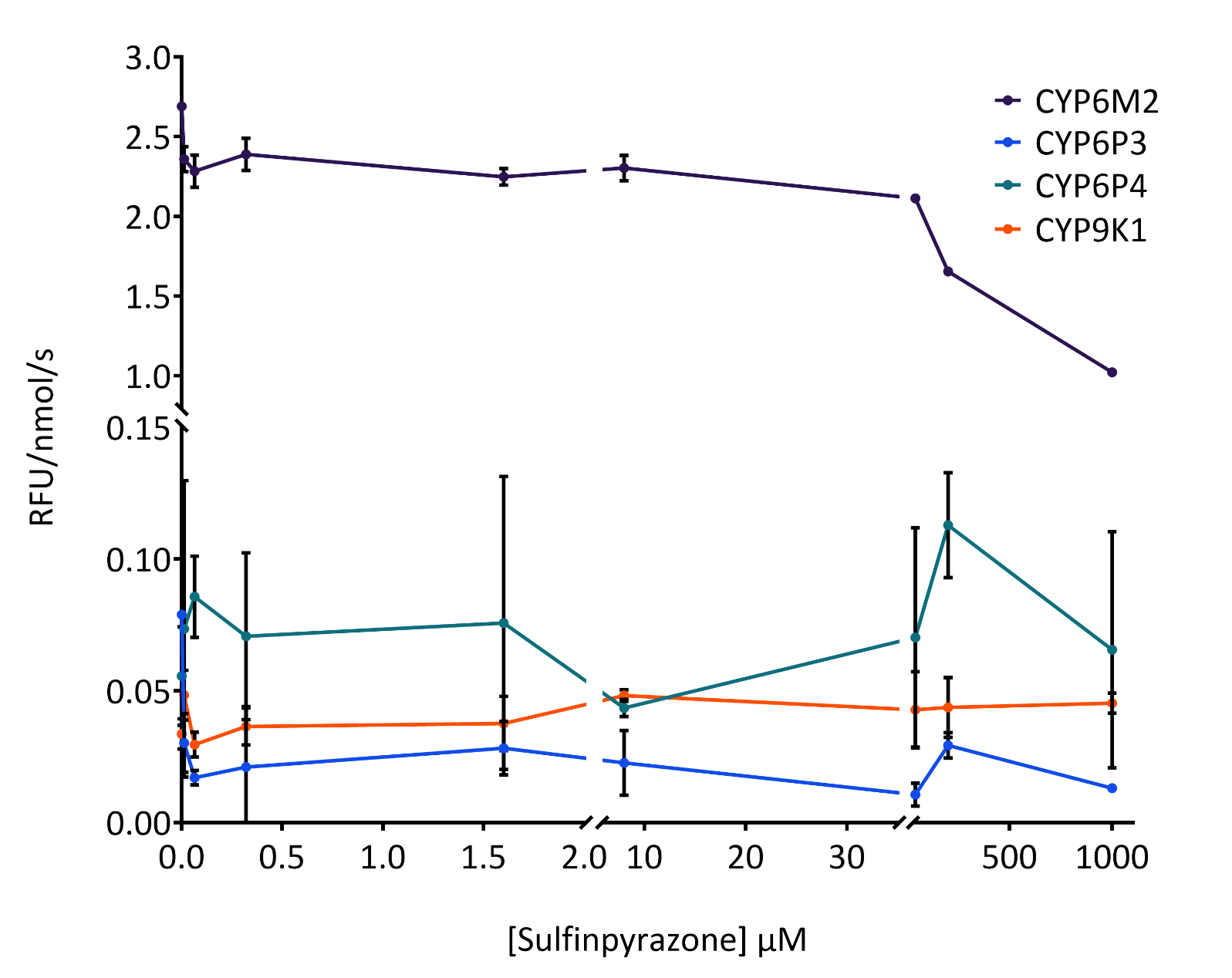


**Figure S5. Investigation of sulfinpyrazone affinity to Cytochrome P450s.** CYP6M2 (purple), 6P3 (blue), 6P4 (teal) and 9K1 (orange) showed no changes in production of fluorescence with increasing doses of sulfinpyrazone demonstrating no affinity of CYP450s towards this compound. The *y-axis* is the relative fluorescence units produced per nmol CYP450 per second (RFU/nmol/s) mortality (%), and the *x-axis* is concentration of sulfinpyrazone (μM) , the data are mean ± SD.
